# *Herba Patriniae* Component Linarin Induces Cell Cycle Arrest and Senescence in Non-Small-Cell Lung Cancer Associated with Cyclin A2 Downregulation

**DOI:** 10.3390/ph19010111

**Published:** 2026-01-08

**Authors:** Wen Xie, Xia Li, Dongmei Huang, Jiana Xu, Minghan Yu, Yanping Li, Qing K. Wang

**Affiliations:** 1 Key Laboratory of Functional and Clinical Translational Medicine, Fujian Province University, Xiamen Medical College, Xiamen 361023, China; 202000010431@xmmc.edu.cn (D.H.); xujiana@xmmc.edu.cn (J.X.); yuminghan@xmmc.edu.cn (M.Y.); 201900010478@xmmc.edu.cn (Y.L.); 2 Fujian Key Laboratory of Neonatal Diseases, Xiamen Children’s Hospital (Children’s Hospital of Fudan University at Xiamen), Xiamen 361006, China; yushengaaaa@163.com; 3 Center for Human Genome Research, Key Laboratory of Molecular Biophysics of the Ministry of Education, College of Life Science and Technology, Huazhong University of Science and Technology, Wuhan 430074, China

**Keywords:** *Herba Patriniae*, non-small-cell lung cancer, Linarin, cell cycle, cellular senescence

## Abstract

**Background**: Non-small-cell lung cancer (NSCLC) remains a major therapeutic challenge due to its high incidence and mortality. *Herba Patriniae* (HP), a traditional Chinese medicine, has long been used for respiratory disorders and exhibits anti-cancer potential. However, the therapeutic effects of HP on NSCLC and the underlying mechanisms have not been fully elucidated. **Methods**: Network pharmacology was applied to identify the core active components of HP and their potential targets in NSCLC. The anti-cancer effects of the core HP component Linarin on the malignant phenotypes of NSCLC cells were characterized using Tumor Protein P53 (p53) wild-type A549 and p53-null H1299 cell lines with Cell Counting Kit-8 (CCK-8), EdU fluorescence staining, colony formation, apoptosis analysis, cell cycle analysis, and senescence-associated β-galactosidase (SA-β-gal) staining, together with molecular docking and Western blotting analyses. **Results**: Network pharmacology analysis identified Linarin as the core active component of HP and screened out six hub targets, including Cyclin Dependent Kinase 1/4 (CDK1/4), Cyclin A2/B1 (CCNA2/B1), and Checkpoint Kinase 1/2 (CHEK1/2), which were found to be mainly enriched in cell cycle and senescence pathways. In vitro assays showed that Linarin dose-dependently (0–200 μM) inhibited NSCLC cell proliferation, induced G0/G1 phase arrest, and promoted cellular senescence and apoptosis in both cell lines, irrespective of p53 status. Molecular docking confirmed strong binding affinities between Linarin and the hub targets, and Western blotting confirmed that Linarin downregulated CCNA2/B1 and CHEK1. **Conclusions**: This study demonstrates that Linarin, the core active component of HP, exerts potent anti-NSCLC effects by inducing G0/G1 arrest, senescence, and apoptosis. These effects are associated with the downregulation of key cell cycle regulators, including CCNA2/B1 and CHEK1. Together, these findings highlight the potential of Linarin as a promising therapeutic option for NSCLC.

## 1. Introduction

Lung cancer is one of the most common malignant tumors in humans, and remains a leading cause of cancer-related mortality worldwide. In 2022, nearly 2.5 million new cases of lung cancer and over 1.8 million related deaths were reported globally, accounting for 12.4% of all cancer diagnoses and 18.7% of cancer-related deaths [1]. Lung cancer is generally classified into non-small-cell lung cancer (NSCLC) and small-cell lung cancer (SCLC), with NSCLC comprising approximately 85% of all cases [2]. The most common subtypes of NSCLC include lung adenocarcinoma (LUAD), lung squamous cell carcinoma (LUSC), and, less frequently, large-cell carcinoma [2]. Conventional treatment strategies such as surgery combined with preoperative or postoperative adjuvant chemotherapy and radiotherapy have long been applied to improve the therapeutic rates and prolong survival in NSCLC patients [3]. More recently, targeted therapies, along with antibody-drug conjugates and bispecific antibodies, have been widely introduced into clinical practice. In addition, novel therapeutic modalities such as T-cell engineering, cellular therapies, and cancer vaccines are emerging as promising treatment options [4]. Despite these advances, challenges—including drug resistance, treatment-related toxicity, and high costs—continue to adversely affect patient prognosis and quality of life. Therefore, there remains an urgent need to explore complementary and integrative approaches for NSCLC treatment [5]. In recent years, increasing attention has been given to the role of traditional Chinese medicine (TCM) in lung cancer prevention and management, with accumulating evidence suggesting its potential to alleviate clinical symptoms and related complications, thereby improving the life quality of lung cancer patients [5].

*Herba Patriniae* (HP), also known as “Bai Jiang Cao,” was first recorded in the *Shen Nong’s Herbal Classic*. In China, it has long been used as a traditional herbal medicine while also serving as a health-promoting vegetable and tea product in daily life. Historically, HP was prescribed for conditions such as intestinal carbuncle, lung carbuncle, gynecological epigastric pain, postpartum blood stasis, and eczema. Modern pharmacological studies have revealed that HP exhibits a broad spectrum of biological activities, including anti-cancer, anti-inflammatory, antimicrobial, antioxidant, sedative, hypnotic, anti-angiogenic, and hypoglycemic effects [6,7,8,9]. Experimental studies have demonstrated that ethanol extracts of HP induce apoptosis in colorectal cancer (CRC) cells, suppress tumor proliferation, and inhibit angiogenesis [8,10]. Furthermore, HP extracts and its active component Asperglaucide significantly downregulate the expression of Epidermal Growth Factor Receptor (EGFR), Phosphoinositide 3-Kinase (PI3K), and Protein Kinase B (AKT/PKB) in HCT116 cells and inhibit cell migration [11]. Both the extract of HP and another active component, Isovitexin, promote apoptosis, and inhibit CRC cell proliferation in a dose-dependent manner through activation of the Tumor Protein P53 (p53) signaling pathway [12], further supporting the anti-tumor potential of HP. Beyond CRC, HP extracts have also demonstrated cytotoxicity against various other cancer cell types, including nasopharyngeal carcinoma, cervical cancer, lung cancer, melanoma, leukemia, and fibrosarcoma [13,14,15]. Despite the established anti-cancer potential of HP, research focusing on NSCLC remains very limited. Although one study reported the cytotoxicity of the HP extract Patrinia-glycoside B-II against A549 lung cancer cells [15], the specific mechanisms through which HP acts in NSCLC are still unclear. Notably, in TCM theory, HP has been applied to treat lung carbuncle, a historical classification that encompasses what is now known as lung cancer [5]. Its traditional actions are highly consistent with modern pharmacological evidence: the described effects of clearing heat, detoxifying, and resolving carbuncles directly align with the observed anti-tumor activity [6]. This potential is further supported by several patents for HP’s use in respiratory diseases, including lung cancer, chronic obstructive pulmonary disease, tuberculosis, and pneumonia [6]. However, the key component(s) remains to be identified. Linarin (acacetin-7-O-rutinoside), a natural flavonoid glycoside, has been identified as a bioactive constituent not only in HP but also in a variety of other medicinal plants, most notably those from the Asteraceae, Lamiaceae, and Scrophulariaceae families [16]. It exhibits a wide spectrum of pharmacological activities, including anti-inflammatory, neuroprotective, and anti-cancer effects [16]. Regarding research on Linarin in NSCLC, Seo et al. reported that Linarin exhibits anti-proliferative activity in A549 cells subjected to 24 h serum-free pretreatment [17]. Jung et al. reported that Linarin inhibits radiation-induced A549 metastasis associated with regulating NF-κB/MMP-9 signaling pathways [18].

Although the therapeutic effects of HP in cancer and respiratory diseases have been increasingly recognized, its potential and specific mechanisms in the treatment of lung cancer remain poorly understood. A comprehensive understanding of its pharmacological actions in lung cancer is therefore needed. In this study, we employed a network pharmacology approach to identify the core active components of HP for the treatment of NSCLC. Based on the Gene Expression Omnibus (GEO) database and pharmacological analysis, we further investigated the potential targets and pathways of HP against NSCLC. Experimental studies were also conducted to explore the pharmacological effects and underlying mechanisms of HP in NSCLC. By integrating network pharmacology with experimental validation, this study provides new insights for understanding the role of HP and its core components in NSCLC, thereby offering a theoretical basis for future experimental research and clinical applications.

## 2. Results

### 2.1. Screening of Active Components and Potential Targets of HP

**Figure 1:**
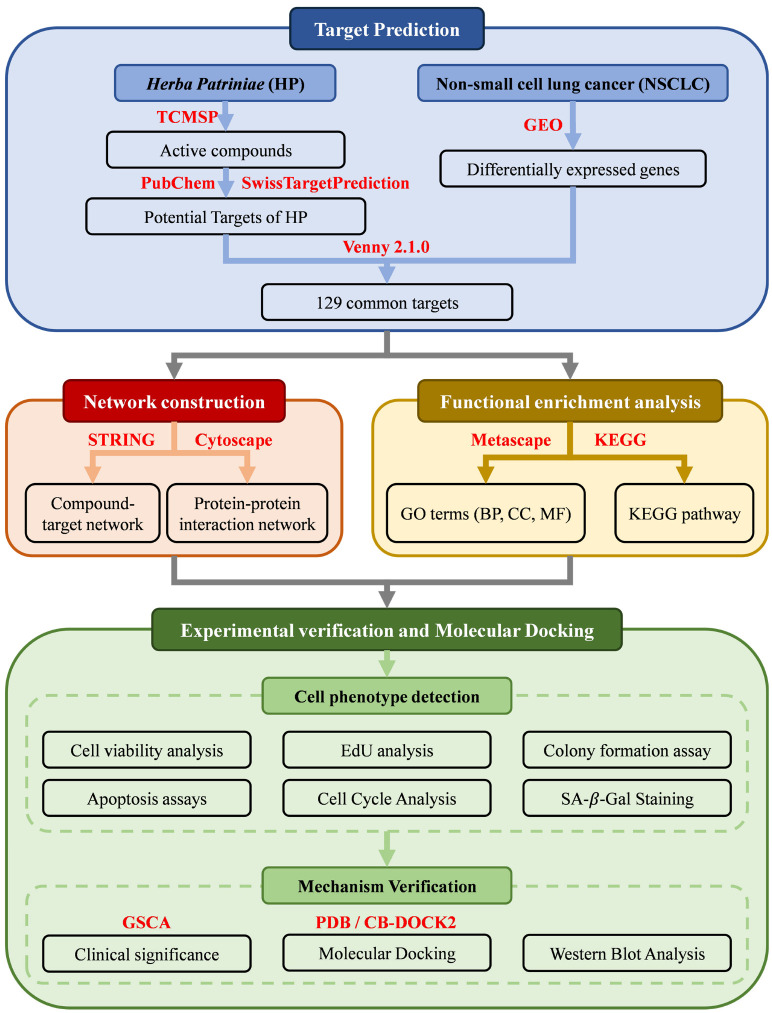
Flow diagram of study search. Abbreviations: HP, *Herba Patriniae*; NSCLC, non-small-cell lung cancer; GEO, Gene Expression Omnibus; GO, Gene Ontology; BP, biological processes; CC, cellular components; MF, molecular function; KEGG, Kyoto Encyclopedia of Genes and Genomes; SA-β-gal, senescence-associated β-galactosidase.

A schematic diagram of the study workflow is shown in Figure 1. A total of 13 active components of HP were retrieved from the TCMSP database using the thresholds of OB (Oral bioavailability) ≥ 30% and DL (Drug-likeness) ≥ 0.18 (Table A1). Potential targets of these components were predicted using the SwissTargetPrediction database, yielding 497 unique targets.

### 2.2. Identification of NSCLC-Related Targets

**Figure 2:**
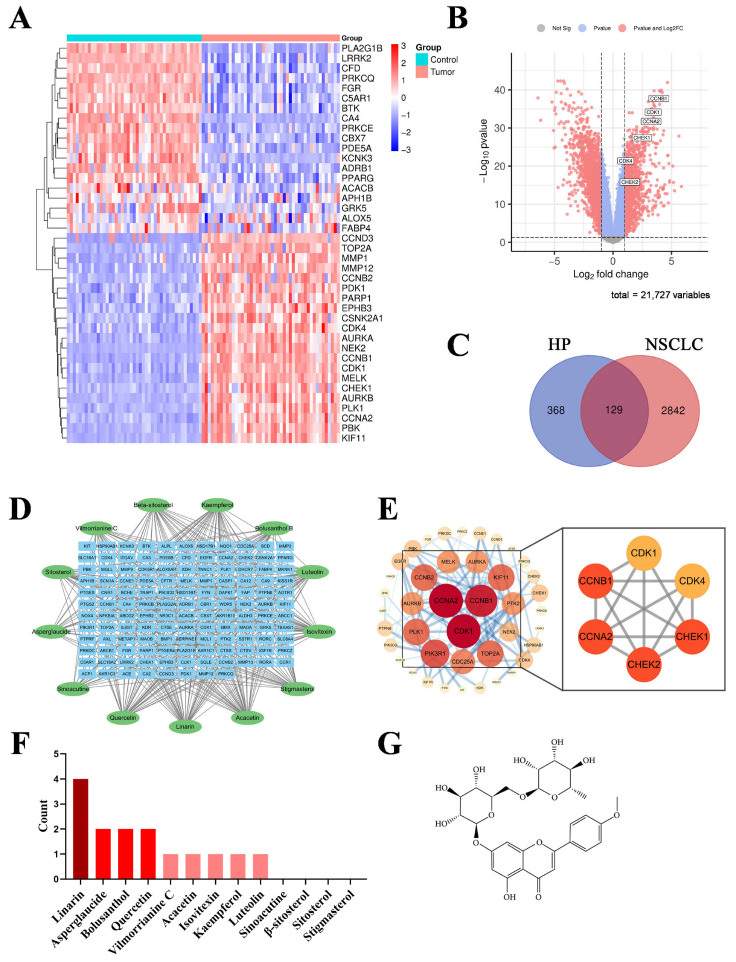
Network pharmacology analysis identifies Linarin as the core active component of HP against NSCLC. (**A**) Heatmap of 40 representative genes. These genes were identified as differentially expressed genes (DEGs) in NSCLC (GSE18842), overlapped with HP’s predicted targets, and selected as the top-ranked by *p*-value for visualization; (**B**) Volcano plot of the DEGs, with the six upregulated cell cycle-related hub genes, including Cyclin Dependent Kinase 1/4 (CDK1/4), Cyclin A2/B1 (CCNA2/B1) and Checkpoint Kinase 1/2 (CHEK1/2), highlighted by the white box. These DEGs are enriched in cell cycle regulation pathway, aligning with the core phenotype investigated in this study; (**C**) Venn diagram identifying 129 common targets at the intersection of HP’s potential targets and NSCLC-related DEGs, highlighting the potential therapeutic targets of HP against NSCLC; (**D**) The component-target network illustrates the connections between the 13 active components of HP (green ellipses) and the 129 common targets (blue rectangles) against NSCLC; (**E**) Protein–protein interaction (PPI) network of the 129 common targets. The six central hub genes identified by the CytoNCA plugin are key regulators of cell cycle progression. Node size and color depth are proportional to the value of the degree; (**F**) Ranking of the 13 candidate active components based on the number of associated hub targets. Linarin is prioritized for experimental validation as it shows the highest connectivity to the hub targets in the network. (**G**) Chemical structure of Linarin [16].

Bioinformatics approaches were employed to explore potential therapeutic targets of NSCLC. The GSE18842 dataset was obtained from the GEO database, which included microarray data from 46 NSCLC tumor samples and 45 control samples. A total of 2971 differential expressed genes (DEGs) were identified under the criteria of adj. P.Val < 0.05 and |logFC| ≥ 1. The volcano plot and heatmap are presented in Figure 2A,B, respectively.

### 2.3. Construction and Analysis of Protein–Protein Interaction (PPI) Network

The intersection between NSCLC-related targets and HP potential targets was obtained using the Draw Venn Diagram tool, resulting in 129 common targets (Figure 2C), which were considered as potential therapeutic targets of HP against NSCLC. A component-target network was then constructed based on 13 active components of HP and 129 common targets using Cytoscape 3.10.0 (Figure 2D). These targets were subsequently imported into the STRING database to construct a PPI network to identify core targets, which was visualized with Cytoscape (Figure 2E). The top 6 hub targets, including Cyclin Dependent Kinase 1/4 (CDK1/4), Cyclin A2/B1 (CCNA2/B1) and Checkpoint Kinase 1/2 (CHEK1/2), were identified using the CytoNCA plugin (Figure 2E, Table A2). Further analysis revealed that the number of hub targets associated with Linarin, identified as a major active component in HP via Ultra Performance Liquid Chromatography-Mass Spectrometry (UPLC-MS) analysis [12], was significantly higher than that of other components (Figure 2F), suggesting that Linarin is the most likely therapeutic component of HP for NSCLC. The chemical structure of Linarin is presented in Figure 2G [16].

### 2.4. GO and KEGG Pathway Enrichment Analysis of Common Targets

**Figure 3:**
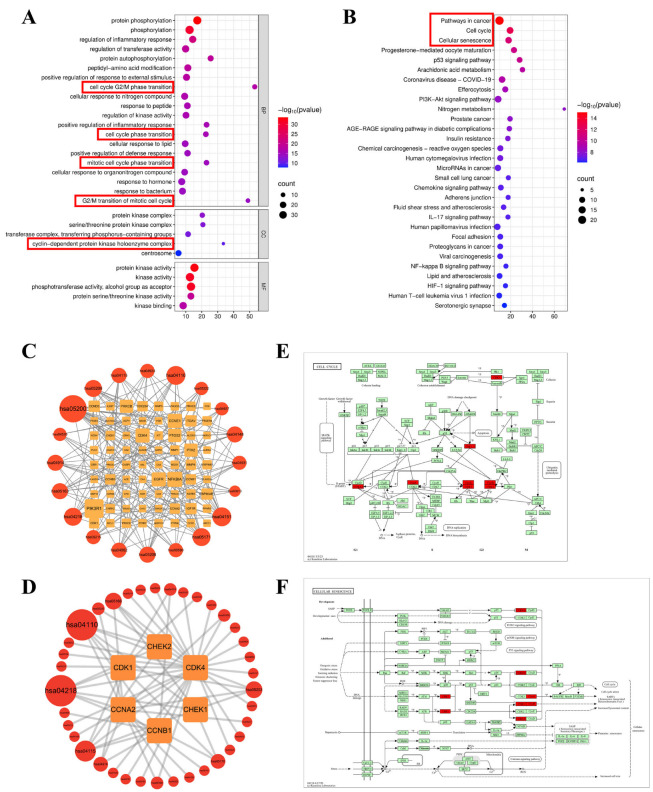
GO and KEGG pathway enrichment analysis. (**A**) GO enrichment analysis of the 129 common targets, categorized into Biological Process (BP), Cellular Component (CC), and Molecular Function (MF). The targets are significantly enriched in cell cycle-related processes (highlighted by the red box), suggesting a potential mechanism of action for HP against NSCLC; (**B**) KEGG pathway enrichment analysis. The common targets are predominantly enriched in cancer-related pathways, with ‘Cell cycle’ and ‘Cellular senescence’ being the most significant, directly guiding the subsequent experimental focus; (**C**,**D**) Network visualizations of the connections between significantly enriched KEGG pathways and targets. Red circles represent KEGG pathways, and orange rectangles represent the common targets (**C**) or the six hub targets (**D**). The size of each node is proportional to its number of connections within the network. Larger nodes represent more highly connected and functionally important elements. hsa05200: Pathways in cancer, hsa04110: Cell cycle, hsa04218: Cellular senescence; (**E**,**F**) Direct mapping of the six hub targets (highlighted in red) onto the KEGG pathway diagrams for ‘Cell cycle’ (**E**) and ‘Cellular senescence’ (**F**).

Gene Ontology (GO) functional enrichment analysis of the 129 common targets was performed using the Metascape database. In terms of biological processes (BP), the targets were primarily involved in protein phosphorylation, regulation of inflammatory responses, regulation of transferase activity, peptidyl-amino acid modification, positive regulation of responses to external stimulus, and cell cycle G2/M phase transition. For cellular components (CC), the targets were mainly enriched in structures such as protein kinase complexes, transferase complexes, and cyclin-dependent protein kinase holoenzyme complexes. Regarding molecular function (MF), the targets were significantly enriched in protein kinase activity and binding, as well as phosphotransferase activity (Figure 3A). Kyoto Encyclopedia of Genes and Genomes (KEGG) pathway enrichment analysis revealed that the hub targets of HP against NSCLC were predominantly associated with multiple cancer-related pathways, including pathways in cancer, cell cycle, cellular senescence, the p53 signaling pathway, prostate cancer, chemical carcinogenesis, microRNAs in cancer, and SCLC (Figure 3B). The common targets involved in each pathway are shown in Figure 3C. Together, these analyses suggest that the potential mechanism of HP against NSCLC is closely linked to cell cycle regulation (highlighted in the red box (Figure 3A–C)).

Subsequent KEGG analysis of the 6 hub targets indicated that the most significantly enriched pathways were cell cycle and cellular senescence pathways (Figure 3D). The 6 hub targets were involved in both pathways (Figure 3D–F). In the KEGG pathway network diagram, the red highlights clearly indicate the specific positions of these 6 hub targets within the key pathways (Figure 3E,F). In summary, HP may regulate the aforementioned hub targets to induce cell cycle arrest and promote cellular senescence, ultimately inhibiting the progression of NSCLC.

### 2.5. Anti-Cancer Activities of Linarin in A549 Cells

**Figure 4:**
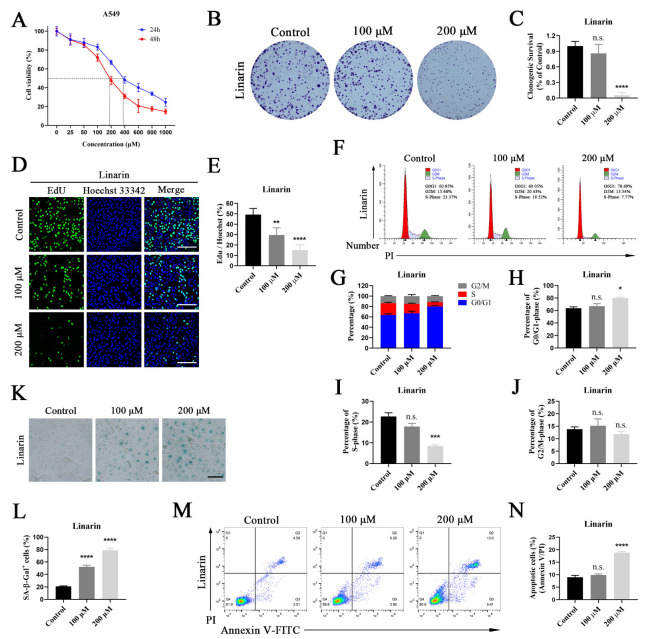
Effects of Linarin on the malignant phenotypes of A549 cells. (**A**) A549 cells were treated with the indicated doses of Linarin for 24 and 48 h to assess cell viability; (**B**) The impact of Linarin on the colony formation of A549 cells; (**C**) Quantification of colony formation assay; (**D**) A549 cells were treated with the indicated doses of Linarin for 24 h, followed by EdU experiments to evaluate cell proliferation (Scale bar: 200 μm). More than 100 cells were counted in each sample; (**E**) Quantification of EdU assay. Control = 49.27 ± 3.37%, 100 μM = 29.56 ± 3.98%, 200 μM = 15.13 ± 2.90%; (**F**) Flow cytometric analysis of cell cycle in A549 cells treated with the indicated doses of Linarin for 24 h. The percentages of cells in G0/G1, S, and G2/M phases are indicated within each panel; (**G**–**J**) Quantification of cell cycle distribution; (**G**) The distribution of cells across all three phases of the cell cycle; (**H**) The percentage of cells in the G0/G1 phase (Control = 63.47 ± 2.33%, 100 μM = 66.92 ± 3.90%, 200 μM = 79.77 ± 1.60%); (**I**) The percentage of cells in the S phase (Control = 22.70 ± 1.78%, 100 μM = 17.87 ± 1.51%, 200 μM = 8.40 ± 0.63%); (**J**) The percentage of cells in the G2/M phase (Control = 13.83 ± 0.91%, 100 μM = 15.21 ± 2.74%, 200 μM = 11.83 ± 1.05%); (**K**) SA-β-gal staining of A549 cells (Scale bar: 100 μm). The blue granular staining indicates senescent cells. More than 100 cells were counted in each sample; (**L**) Quantification of SA-β-gal staining. Control = 20.68 ± 0.50%, 100 μM = 52.21 ± 1.54%, 200 μM = 78.87 ± 2.04%; (**M**) Apoptosis was measured using flow cytometric analysis. Cell populations were distinguished as follows: viable (Annexin V^−^/PI^−^), early apoptotic (Annexin V^+^/PI^−^), late apoptotic (Annexin V^+^/PI^+^), and necrotic (Annexin V^−^/PI^+^); (**N**) Percentage of apoptotic cells. The apoptotic cell population is defined as the combination of early and late apoptotic cells. Control = 8.95 ± 0.44%, 100 μM = 9.87 ± 0.25%, 200 μM = 18.82 ± 0.29%. Data are represented as mean ± SEM (n = 3). Statistical significance: n.s., not significant; * *p* < 0.05, ** *p* < 0.01, *** *p* < 0.001, **** *p* < 0.0001.

To evaluate the inhibitory effect of Linarin on NSCLC cell proliferation, p53 wild-type A549 cells were treated with varying concentrations of Linarin, and cell viability was assessed by Cell Counting Kit-8 (CCK-8) assay. Linarin significantly inhibited the viability of A549 cells in a dose-dependent manner (Figure 4A). The colony formation assay further demonstrated that Linarin suppressed the clonogenic ability of A549 cells (Figure 4B,C). EdU fluorescence staining, which could measure DNA replication activity, revealed that both concentrations of Linarin significantly reduced the proportion of proliferating A549 cells (Figure 4D,E). These results indicate that Linarin effectively inhibits A549 cell proliferation.

KEGG analysis suggested that the anti-NSCLC effect of HP was closely associated with cell cycle and senescence pathways (Figure 3); thus, we further investigated the effect of Linarin on the cell cycle distribution of A549 cells. Linarin treatment decreased the proportion of S-phase cells and induced G0/G1 phase arrest (Figure 4F–J). Since permanent withdrawal from the cell cycle can induce senescence and apoptosis [19], we subsequently examined the effect of Linarin on cellular senescence. Senescence-associated β-galactosidase (SA-β-gal) staining showed that Linarin significantly increased the proportion of SA-β-gal-positive cells, suggesting that it promotes senescence in A549 cells (Figure 4K,L). Annexin V/PI staining further demonstrated that Linarin significantly increased apoptosis of A549 cells (Figure 4M,N). Overall, Linarin effectively inhibited the proliferation of A549 cells and exerted anti-tumor effects by inducing G0/G1 phase arrest, promoting cellular senescence and triggering apoptosis.

### 2.6. Anti-Cancer Activities of Linarin in p53-Null H1299 Cells

**Figure 5:**
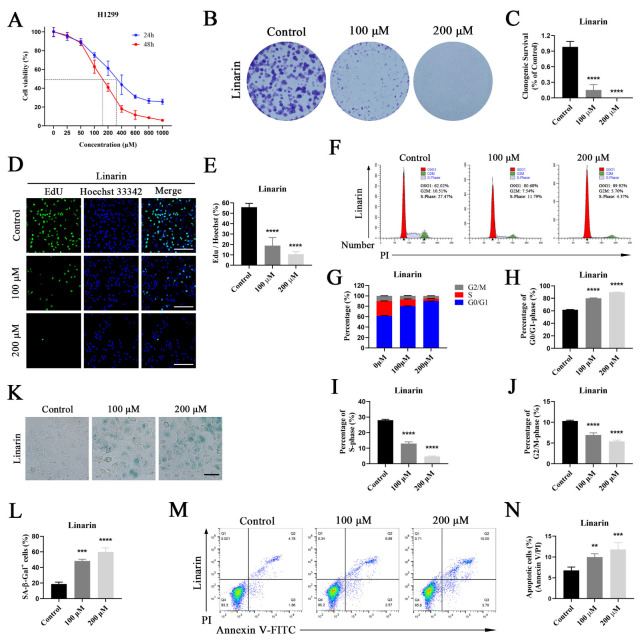
Effects of Linarin on the malignant phenotypes of H1299 cells. (**A**) H1299 cells were treated with the indicated doses of Linarin for 24 and 48 h to assess cell viability; (**B**) The impact of Linarin on the colony formation of H1299 cells. (**C**) Quantification of colony formation assay; (**D**) H1299 cells were treated with the indicated doses of Linarin for 24 h, followed by EdU experiments to evaluate cell proliferation (Scale bar: 200 μm). More than 100 cells were counted in each sample; (**E**) Quantification of EdU assay. Control = 55.87 ± 2.11%, 100 μM = 18.88 ± 4.45%, 200 μM = 10.62 ± 1.39%; (**F**) Flow cytometric analysis of cell cycle in H1299 cells treated with the indicated doses of Linarin for 24 h. The percentages of cells in G0/G1, S, and G2/M phases are indicated within each panel; (**G**–**J**) Quantification of cell cycle distribution; (**G**) The distribution of cells across all three phases of the cell cycle; (**H**) The percentage of cells in the G0/G1 phase (Control = 61.64 ± 0.25%, 100 μM = 80.05 ± 0.39%, 200 μM = 89.88 ± 0.10%); (**I**) The percentage of cells in the S phase (Control = 28.04 ± 0.31%, 100 μM = 13.00 ± 0.63%, 200 μM = 4.69 ± 0.23%); (**J**) The percentage of cells in the G2/M phase (Control = 10.32 ± 0.10%, 100 μM = 6.95 ± 0.30%, 200 μM = 5.43 ± 0.16%); (**K**) SA-β-gal staining of H1299 cells (Scale bar: 100 μm). The blue granular staining indicates senescent cells. More than 100 cells were counted in each sample; (**L**) Quantification of SA-β-gal staining. Control = 18.65 ± 1.42%, 100 μM = 48.45 ± 1.18%, 200 μM = 59.91 ± 3.06%; (**M**) Apoptosis was measured using flow cytometric analysis. Cell populations were distinguished as follows: viable (Annexin V^−^/PI^−^), early apoptotic (Annexin V^+^/PI^−^), late apoptotic (Annexin V^+^/PI^+^), and necrotic (Annexin V^−^/PI^+^); (**N**) Percentage of apoptotic cells. The apoptotic cell population is defined as the combination of early and late apoptotic cells. Control = 6.80 ± 0.46%, 100 μM = 9.99 ± 0.46%, 200 μM = 11.80 ± 0.95%. Data are represented as mean ± SEM (n = 3). Statistical significance: ** *p* < 0.01, *** *p* < 0.001, **** *p* < 0.0001.

As a critical tumor suppressor, p53 plays a key role in cell cycle regulation and senescence by modulating regulatory factors such as Cyclin Dependent Kinase Inhibitor 1A (p21), Cyclin D1, CDK4, and Cyclin Dependent Kinase 2 (CDK2) [20,21]. To determine whether the inhibitory effect of Linarin on NSCLC depends on p53, the above experiments were repeated in p53-null H1299 cells. The CCK-8 assay showed that Linarin inhibited the viability of H1299 cells in a dose-dependent manner (Figure 5A). Similarly, colony formation and EdU assays indicated that Linarin significantly suppressed both the clonogenic ability and proliferative activity (Figure 5B–E). Flow cytometry analysis revealed that Linarin induced G0/G1 arrest in a dose-dependent manner (Figure 5F–J). SA-β-gal staining demonstrated that Linarin significantly promoted senescence of H1299 cells (Figure 5K,L). Annexin V/PI staining confirmed that Linarin also markedly induced apoptosis of H1299 cells (Figure 5M,N). These findings indicated that Linarin effectively inhibited NSCLC cell proliferation, induced G0/G1 arrest, and promoted senescence and apoptosis in the absence of p53, suggesting that its functions were independent of the p53 pathway.

### 2.7. Clinical Significance of Hub Genes in NSCLC

**Figure 6:**
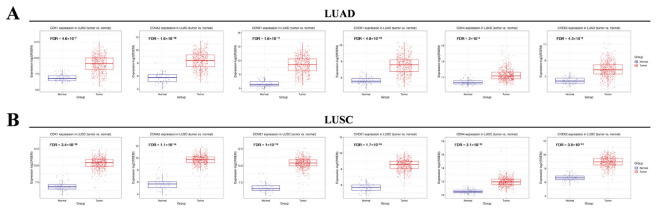
The expression levels of CDK1/4, CCNA2/B1 and CHEK1/2 in (**A**) lung adenocarcinoma (LUAD) and (**B**) lung squamous cell carcinoma (LUSC) tissues based on the TCGA dataset.

Based on The Cancer Genome Atlas (TCGA) database, we analyzed the expression of the 6 hub genes in 517 LUAD and 503 LUSC samples. The expression levels of these genes were significantly higher in both LUAD and LUSC tissues compared with normal tissues (Figure 6), suggesting their potential as therapeutic targets in NSCLC.

### 2.8. Verification of Component-Target Interactions

**Figure 7:**
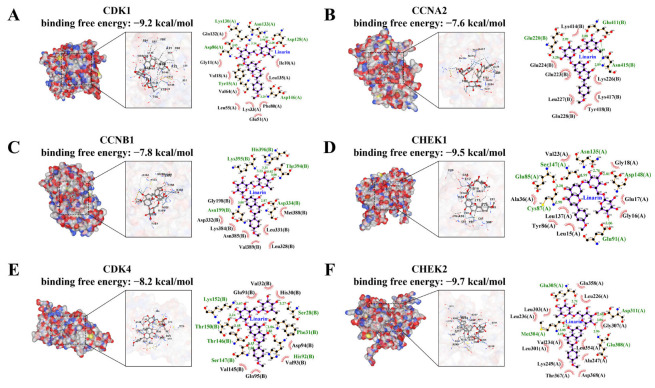
Molecular docking 3D map and 2D pose view of Linarin and hub protein targets interactions. (**A**) Linarin and CDK1, (**B**) Linarin and CCNA2, (**C**) Linarin and CCNB1, (**D**) Linarin and CHEK1, (**E**) Linarin and CDK4, (**F**) Linarin and CHEK2. The left of each panel represents molecular docking 3D map of Linarin and hub proteins analyzed by CB-DOCK2, highlighting the binding posture with the lowest energy. The right of each panel represents 2D interacting forces for Linarin-hub protein complexes provided with the Ligplot+ program. Hydrogen-bonding interactions highlighted by the green dashed line and hydrophobic interactions highlighted by the arc-shaped eyelash pattern.

To evaluate the binding affinity between Linarin and the hub target genes, molecular docking analysis was performed using CB-DOCK2. According to the Vina scoring criterion, a binding free energy of ≤−5.0 kcal/mol indicates favorable ligand-receptor interaction [22,23]. The results demonstrated that the binding free energies between Linarin and 6 hub targets were all below −7 kcal/mol, suggesting a strong binding affinity with each target (Figure 7). The molecular docking results are visualized in Figure 7.

### 2.9. Linarin Inhibits Cell Cycle-Related Protein Expression

**Figure 8:**
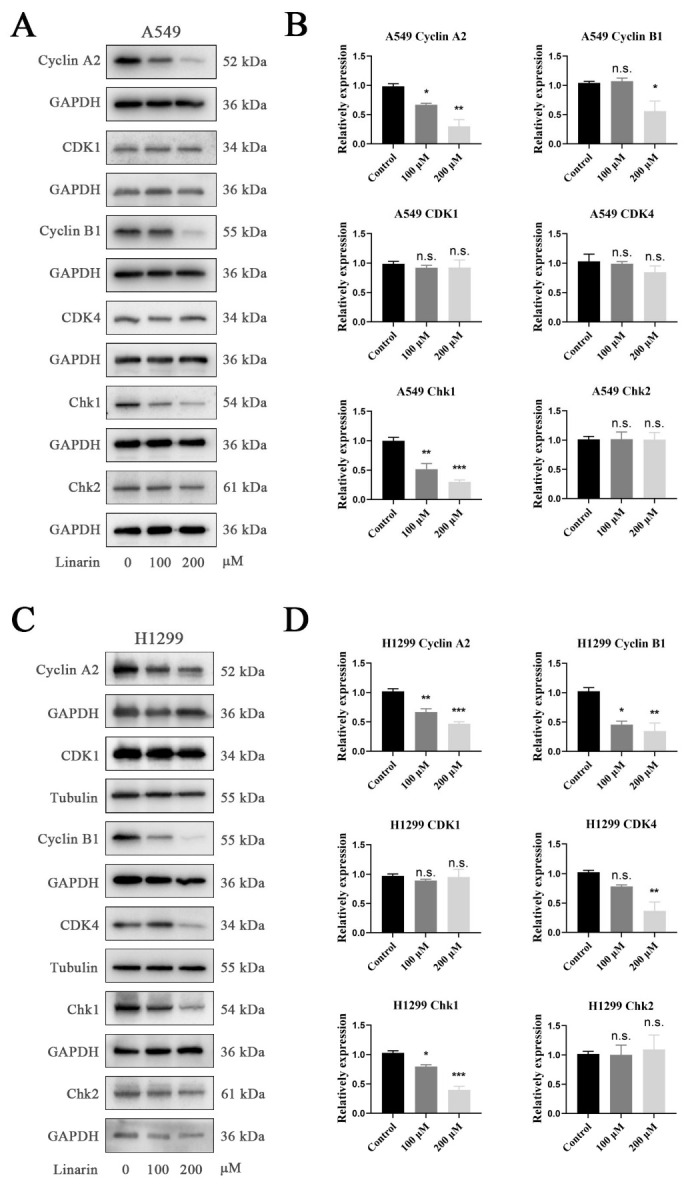
Linarin downregulates cell cycle-related hub proteins in A549 and H1299 cells. (**A**,**C**) Representative Western blot images showing the expression of key cell cycle proteins (CDK1/4, CCNA2/B1 (Cyclin A2/B1), CHEK1/2 (Chk1/2)) in A549 cells (**A**) and H1299 cells (**C**) after treatment with Linarin (0, 100, 200 μM) for 24 h. GAPDH or α-Tubulin served as the loading control. (**B**,**D**) Quantitative densitometric analysis of the protein bands presented in panel A (**B**) and panel C (**D**), respectively. Data are represented as mean ± SEM (n = 3). Statistical significance: n.s., not significant; * *p* < 0.05, ** *p* < 0.01, *** *p* < 0.001.

To further elucidate the underlying molecular mechanisms of Linarin treatment, we examined hub target protein expression in A549 and H1299 cells following Linarin treatment using Western blotting. In A549 cells, Linarin significantly downregulated the protein levels of Cyclin A2 (encoded by *CCNA2*) and Chk1 (encoded by *CHEK1*) at both 100 μM and 200 μM concentrations, and Cyclin B1 (encoded by *CCNB1*) at 200 μM (Figure 8A,B). Similarly, in H1299 cells, Linarin markedly inhibited the expression of Cyclin A2/B1 and Chk1 at both concentrations, while CDK4 expression was suppressed only at the higher concentration (Figure 8C,D). Collectively, these findings demonstrate that Linarin treatment induces G0/G1 arrest and promotes cellular senescence. These effects are associated with the downregulation of critical cell cycle regulators, including Cyclin A2/B1 and Chk1.

## 3. Discussion

Lung cancer, particularly NSCLC, remains one of the most prevalent malignant tumors with the highest incidence and mortality rates worldwide. Its complex pathogenesis poses a major challenge for clinical treatment [1,2]. TCM, characterized by its multi-component and multi-target mechanisms along with favorable safety profiles, has demonstrated unique advantages in cancer therapy [5]. As a traditional herbal medicine, HP has been empirically proven to be effective in treating various respiratory diseases and exhibits promising potential as an anti-cancer adjuvant [6,24]. However, its active components and the specific therapeutic efficacy and molecular mechanisms in NSCLC remain unclear. This study integrated network pharmacology analysis with experimental validation to systematically show that Linarin is a major active component of HP, and exerts anti-tumor effects, accompanied by downregulation of key cell cycle regulators, induction of G0/G1 arrest and cellular senescence, thereby providing new molecular evidence and mechanism for the anti-NSCLC activity of HP.

Our network pharmacology analysis identified 13 active components in HP and predicted 129 potential targets for NSCLC therapy. The PPI network pinpointed 6 key hub targets, including CDK1/4, CCNA2/B1 and CHEK1/2. Among 13 active components, Linarin emerged as the core active component, which interacted with the highest number of hub targets. Linarin, a natural flavonoid, exhibits diverse pharmacological activities, including anti-cancer effects [25,26]. Previous studies have demonstrated that Linarin induces apoptosis, inhibits proliferation and migration of glioma cells via the NF-κB/p65 and p53 signaling pathways [27]. Additionally, Linarin significantly enhances TRAIL (tumor necrosis factor-related apoptosis-induced ligand)-induced cytotoxicity and apoptosis by regulating generation of reactive oxygen species (ROS) [28], further highlighting its tumor-suppressing potential. Through multi-level analyses, we identified cell cycle regulation and cellular senescence as the core mechanisms underlying the anti-tumor effects of HP. Experimental studies further confirmed that Linarin significantly inhibited A549 cell proliferation, induced G0/G1 arrest, and promoted cellular senescence. Notably, Linarin exhibited even stronger inhibitory effects in p53-null H1299 cells, indicating that its anti-tumor activity is independent of the classical p53 pathway.

At the molecular level, the hub target genes and proteins identified in this study were significantly overexpressed in NSCLC tissues, underscoring their importance in NSCLC pathogenesis and potential as prognostic markers and therapeutic targets. Molecular docking and Western blotting analyses suggested that Linarin exhibited strong binding affinities with hub targets such as Cyclin A2/B1 and Chk1, and significantly downregulated their protein expression. The downregulation of Cyclin A2 is particularly critical for halting the G1/S transition [19]. By inducing G0/G1 phase arrest, Linarin may prevent the initiation of DNA replication, ultimately leading to cellular senescence and apoptosis. As a master regulator of the DNA damage response, the suppression of Chk1 creates a vulnerable state in cancer cells [19]. Although traditionally recognized for its role in cell cycle checkpoints, inhibition of Chk1 also leads to replication stress and the accumulation of DNA damage during S-phase. The downregulation of Chk1 by Linarin not only disrupts normal cell cycle progression but may also exacerbate intrinsic replication stress, thereby pushing the cells toward senescence or apoptosis. Although Cyclin B1 is primarily associated with the G2/M transition [19], its downregulation could be a consequence of sustained G1 arrest and a general failure of cell cycle progression. It may also indicate a broader disruption of the cyclin network, thereby reinforcing the cell cycle blockade. In summary, Linarin launches a multi-faceted attack on the cell cycle machinery by simultaneously downregulating Cyclin A2—a key regulator of the G1/S transition—and disabling the Chk1, thereby triggering an irreversible cell cycle exit. Among these, Cyclin A2 appears to be a major target of Linarin. Cyclin A2 not only plays a pivotal role in cell cycle progression but is also implicated in tumor invasion and metastasis, making it a potential therapeutic target in cancer [29]. The synergistic effects on Cyclin B1 and Chk1 further underscore the multi-target regulatory advantage of Linarin. As our analysis of the TCGA database confirmed, Cyclin A2/B1 and Chk1 are significantly overexpressed in LUAD and LUSC tissues (Figure 6). This overexpression is often associated with poor prognosis and therapy resistance. Therefore, the ability of Linarin to simultaneously target this set of oncogenic drivers holds significant therapeutic promise.

Interestingly, Linarin exhibited a stronger tumor-suppressive effect in p53-deficient H1299 cells, which may be associated with the downregulation of CDK4. CDK4 phosphorylates and inactivates RB, thereby releasing E2F transcription factors to promote the G1/S transition [30]. Accordingly, the downregulation of CDK4 observed in H1299 cells under high concentrations of Linarin may further enhance G0/G1 arrest. Taken together, the multi-target and pathway-independent characteristics of Linarin highlight its potential as a promising therapeutic candidate for NSCLC, particularly in patients harboring *TP53* mutations.

Our findings regarding the anti-NSCLC effects of Linarin extend the knowledge from previous studies. Seo et al. reported that Linarin inhibits A549 cell proliferation associated with suppressing Akt activation and inducing p27^Kip1^ expression [17]. In addition, Jung et al. demonstrated that Linarin suppresses radiation-induced A549 cell invasion via the NF-κB/MMP-9 pathway [18]. Our study identifies a distinct mode of action for Linarin. Unlike the previously reported Akt or NF-κB pathways, we combined network pharmacology with experimental validation to reveal that Linarin primarily targets the cell cycle machinery by downregulating Cyclin A2/B1 and Chk1. Most importantly, we provide the first evidence that Linarin induces cellular senescence in NSCLC cells and retains robust efficacy in p53-null cells, suggesting broader therapeutic potential for patients with *TP53* mutations. Regarding the effective concentration of Linarin, Seo et al. observed inhibitory effects at 10 μM, while our study utilized concentrations of 100-200 μM. This discrepancy is likely attributable to differences in cell treatment strategies. Seo et al. pretreated cells with serum-free medium for 24 h prior to Linarin treatment [17]. This serum starvation likely placed the cells in a stress-sensitized state, thereby significantly lowering their threshold for Linarin-induced inhibition of proliferation. On the other hand, Jung et al. reported an IC_50_ of 282 μM for A549 cell viability [18], which is consistent with the concentration range used in our cytotoxicity and cell cycle assays. While lower concentrations may be sufficient to suppress A549 cell proliferation under starvation-mimicking condition or inhibit cell migration, higher concentrations appear necessary to trigger widespread cell cycle arrest and senescence, ultimately leading to cell death.

Previous studies have reported the anti-tumor effects of HP; however, our findings provide new insights into the role of HP and its core active component Linarin in suppressing tumor progression, especially NSCLC, through inhibition of the cell cycle and induction of cellular senescence. In addition, other pathways enriched in our KEGG analysis, such as arachidonic acid metabolism [31,32] and the PI3K-Akt signaling pathway [33], have also been implicated in NSCLC pathogenesis. Future studies should therefore investigate whether HP and its active components modulate these pathways, which may facilitate a more comprehensive exploration of novel therapeutic strategies for NSCLC.

The pharmacological profile of Linarin confers it certain advantages for NSCLC therapies. Unlike targeted agents that inhibit a single oncogenic driver (e.g., EGFR or ALK inhibitors), Linarin exerts inhibitory effect on a set of cell cycle regulating genes (*CCNA2*/*B1* and *CHEK1*). This multi-faceted cell cycle disruption could be particularly advantageous in tumors with complex genetic backgrounds or acquired resistance to single-target drugs. Most notably, our finding that Linarin remains highly effective in p53-null H1299 cells suggests its potential utility in the substantial subset of NSCLC patients harboring *TP53* mutations, a population with poorer response to standard chemotherapy [34]. Furthermore, the reported low toxicity of Linarin toward normal cells supports its exploration not only as a monotherapy but also as an adjunct to current treatments [27]. The dual action of Linarin, inducing cell cycle arrest and cellular senescence, suggests a possible synergy for Linarin with other chemotherapeutic agents or immunotherapy in NSCLC. Firstly, the precise arrest at the G0/G1 phase could be strategically exploited to synchronize tumor cells, potentially enhancing the cytotoxicity of G1 phase-specific chemotherapeutic agents in subsequent treatments. Secondly, the Linarin-induced senescent phenotype warrants investigation in the context of modern immunotherapy. While senescence is a terminal cell cycle arrest, senescent cells remain metabolically active and secrete a plethora of factors known as the senescence-associated secretory phenotype (SASP). In an anti-tumor context, a tailored SASP can promote immune clearance by attracting and activating natural killer (NK) cells and T lymphocytes [35]. Therefore, we hypothesize that Linarin pretreatment might remodel the tumor immune microenvironment, potentially making NSCLC tumor cells more responsive to immunotherapy.

While our study demonstrates the anti-NSCLC potential of Linarin, its achievable systemic concentration and safety profile are critical for clinical translation. First, the effective concentrations used in our in vitro experiments (100–200 µM) are substantially higher than the nanomolar-range plasma concentrations reported for Linarin in animal pharmacokinetic studies [36]. Second, Linarin has been reported to inhibit acetylcholinesterase (AChE) [37,38]. Potent AChE inhibition at high doses of Linarin may lead to cholinergic toxicity—characterized by elevated acetylcholine levels that can cause respiratory failure and trigger status epilepticus—an effect resembling organophosphate poisoning [39]. Therefore, achieving high local concentrations of Linarin at tumor sites without significantly compromising AChE activity is crucial for its further development. Potential strategies may include: (1) structural modification of Linarin (e.g., methylation, acylation) to enhance lipophilicity, improve metabolic stability, increase intestinal absorption, and reduce its AChE inhibitory activity [40]; and (2) development of targeted drug delivery systems (e.g., inhalable nanoparticle-based drug delivery system) to achieve high local concentrations in tumors while minimizing systemic exposure [41], thereby lowering AChE inhibition-associated toxicity.

## 4. Materials and Methods

### 4.1. Prediction of Potential Targets of HP

The chemical components of HP were obtained from the TCMSP database (https://www.tcmsp-e.com/load_intro.php?id=43, accessed on 16 October 2024). TCMSP is a powerful online platform that enables the systematic investigation of the multi-component, multi-target mechanisms of traditional Chinese medicine. It serves as a comprehensive repository containing all 499 herbs documented in the Chinese Pharmacopoeia, with detailed data on over 29,000 chemical components [42]. OB (oral bioavailability) is a crucial pharmacokinetic parameter representing the rate and extent of a drug’s absorption into the systemic circulation. DL (drug-likeness) is a qualitative concept that evaluates the structural similarity of a compound to known drugs. The screening criteria for active components were set as OB ≥ 30% and DL ≥ 0.18 as described previously [12,14]. The molecular structures of the selected active components were retrieved from the PubChem Compound Database (https://www.ncbi.nlm.nih.gov/pccompound, accessed on 16 October 2024), a public repository for chemical structures and biological properties, to facilitate the subsequent molecular docking analysis [43]. These structures were then uploaded to the SwissTargetPrediction database (http://www.swisstargetprediction.ch/, accessed on 16 October 2024) to identify putative target genes. SwissTargetPrediction database forecasts targets by cross-referencing the small molecules against an extensive, up-to-date database of bioactive compounds, leveraging both 2D and 3D similarity calculations [44].

### 4.2. Screening of DEGs Between NSCLC and Non-Tumor Samples

The mRNA expression profiles of 46 NSCLC samples and 45 controls (dataset GSE18842) were obtained from the GEO database (https://www.ncbi.nlm.nih.gov/geo/, accessed on 20 October 2024), a public repository for high-throughput gene expression and other functional genomics data [45]. DEGs were identified using the thresholds of *p* < 0.05 and ∣logFC∣ ≥ 1 as potential NSCLC-related targets. A heatmap and volcano plot were generated using the SRplot platform (http://www.bioinformatics.com.cn/, accessed on 20 October 2024) to visualize the results [46].

### 4.3. Construction of PPI Network

The common targets between HP and NSCLC were identified and visualized using the online Venn diagram tool (Draw Venn Diagram—Universiteit Gent, http://bioinformatics.psb.ugent.be/webtools/Venn/, accessed on 22 October 2024). These common targets were then imported into the STRING database (http://string-db.org/, accessed on 22 October 2024) [47] with an interaction score threshold of 0.9. Disconnected nodes were removed. A PPI network was constructed using Cytoscape 3.10.0. Hub targets were identified using the CytoNCA plugin (version 2.1) of Cytoscape based on the parameters Degree, Eigenvector, Betweenness, and Closeness.

### 4.4. GO and KEGG Pathway Enrichment Analyses

The common targets between HP and NSCLC were uploaded to the Metascape database (https://metascape.org/gp/#/main/step1, accessed on 22 October 2024) [48] for GO and KEGG pathway enrichment analyses. Enriched GO terms and KEGG pathways with *p* < 0.01 were considered statistically significant. The results were visualized as bubble plots using the SRplot online platform. In addition, network analysis was performed using Cytoscape 3.10.0 to elucidate the relationships between these pathways and the hub targets.

### 4.5. Cell Culture and Treatment

The A549 (p53 wild-type) and NCI-H1299 (p53-null) NSCLC cell lines were purchased from ZQXZBIO (Shanghai, China). A549 cells were cultured in F-12K medium (ZQXZBIO, China). NCI-H1299 cells were maintained in RPMI-1640 medium (ZQXZBIO, Shanghai, China). All media were supplemented with 10% fetal bovine serum (ZQXZBIO, Shanghai, China) and 1% penicillin/streptomycin mixture (ZQXZBIO, Shanghai, China). All cells were incubated at 37 °C in a humidified atmosphere containing 5% CO_2_.

Linarin (HPLC: 98.02%, HY-N0528) was purchased from MCE (MedChemExpress, Shanghai, China). Unless otherwise specified, the following treatment procedure was applied throughout the study. A stock solution of Linarin was prepared in dimethyl sulfoxide (DMSO) and diluted in culture medium for all experiments. A549 and H1299 cells were treated with various concentrations of Linarin (0, 100, and 200 μM) for 24 h. Cells treated with an equal volume of DMSO served as the negative control (Linarin 0 μM).

### 4.6. Cell Viability Assay

Cell viability was determined using the CCK-8 assay. Cells were seeded into 96-well plates and treated with various concentrations of Linarin for 24 h at 37 °C. After treatment, 10 μL of CCK-8 reagent (Beyotime, Shanghai, China) was added into each well, followed by incubation for 1–4 h. The optical density (OD) at 450 nm was measured using a microplate reader (Molecular Devices, San Jose, CA, USA) to assess cell viability. Linarin (HPLC: 98.02%, HY-N0528) was purchased from MCE (MedChemExpress, Shanghai, China).

### 4.7. Colony Formation Assay

A total of 1000 A549 and H1299 cells were seeded into 6-well plates and cultured for 4 days. The cells were then treated with different concentrations of Linarin for 6 days. After incubation, the cells were washed three times with PBS, fixed with 4% paraformaldehyde for 30 min, and stained with 0.1% crystal violet (Meilun, Dalian, China) for 20 min. Images were captured, and the number of colonies was counted using ImageJ software (version ImageJ 1.54p).

### 4.8. EdU Assay

According to the manufacturer’s instructions, cell proliferation was measured using an EdU Imaging Kit (488) (APExBIO Technology, Houston, TX, USA). Images were captured using a confocal microscope (Leica, Wetzlar, Germany).

### 4.9. Cell Cycle Assay

According to the manufacturer’s instructions, cell cycle distribution was analyzed using a Cell Cycle and Apoptosis Analysis Kit (Yeasen, Shanghai, China) and analyzed by flow cytometry (Beckman Coulter, Brea, CA, USA). Cells were gated to exclude debris based on FSC vs. SSC, followed by singlet gating based on FSC-A vs. FSC-H to exclude doublets and aggregates prior to analysis. Data were analyzed using FlowJo 10.9 software.

### 4.10. Cell Apoptosis Assay

According to the manufacturer’s instructions, apoptosis was detected using an Annexin V-FITC/PI Apoptosis Detection Kit (Yeasen, Shanghai, China) and analyzed by flow cytometry (Beckman Coulter, Brea, CA, USA). Cells were gated to exclude debris based on FSC vs. SSC, followed by singlet gating based on FSC-A vs. FSC-H to exclude doublets and aggregates prior to analysis. Data were analyzed using FlowJo 10.9 software.

### 4.11. SA-β-Gal Staining

According to the manufacturer’s instructions, cellular senescence was evaluated using a β-Galactosidase Staining Kit (Vazyme, Nanjing, China). Briefly, after Linarin treatment, cells were fixed with Fixative solution for 15 min at room temperature, followed by incubation with β-gal staining solution overnight at 37 °C in the dark without CO_2_. Images were captured using an inverted microscope (Zeiss, Oberkochen, Germany).

### 4.12. Molecular Docking Analysis

The 3D structures of target proteins were obtained from the Protein Data Bank (http://www.rcsb.org/, accessed on 15 March 2025) and subsequently processed by removing redundant chains. The molecular structure of Linarin was retrieved from the PubChem database (https://www.ncbi.nlm.nih.gov, accessed on 15 March 2025). The ligand (Linarin) and receptor (target proteins) were submitted to the CB-DOCK2 server (https://cadd.labshare.cn/cb-dock2/index.php, accessed on 15 March 2025) for molecular docking [49]. Default parameters were applied, and the Vina score was obtained. CB-DOCK2 is a free, web-based software server used for protein–ligand blind docking in bioinformatics and pharmaceutical research. It is designed to predict where a ligand might bind to a larger protein structure and how strongly they interact. A Vina score is a quantitative estimate of the binding affinity between a ligand and a protein. A more negative score indicates a stronger and more stable binding interaction. Vina score ≤ −5.0 kcal/mol indicates favorable ligand-receptor interaction [22,23]. The LigPlot+ software (version v.2.3.1) was used to obtain the 2D binding results between Linarin and hub proteins [50].

### 4.13. Western Blotting Analysis

Cells were lysed using RIPA lysis buffer (Beyotime, Shanghai, China) supplemented with 1% protease inhibitors (Epizyme, Shanghai, China). The proteins were then separated by 10% SDS-PAGE and transferred onto a PVDF membranes. Membranes were blocked with 5% non-fat milk in TBST for 1 h at room temperature and then incubated overnight at 4 °C with specific primary antibodies. After washing, membranes were incubated with HRP-conjugated secondary antibodies for 1 h. Protein bands were visualized using an enhanced chemiluminescence (ECL) reagent (Meilun, Dalian, China) and imaged using a ChemiDoc system (Bio-Rad, Hercules, CA, USA). Band intensities were quantified using ImageJ software. Western blotting experiments were performed in triplicate.

The antibodies used in this study are listed as follows: CDK1 (1:2000, db12527, Diagbio, Hangzhou, China), CCNA2 (1:1000, db12205, Diagbio), CCNB1 (1:1000, db13323, Diagbio), CHEK1 (1:1000, db14895, Diagbio), CDK4 (1:1000, db14093, Diagbio), CHEK2 (1:5000, db12037, Diagbio). GAPDH (1:50,000, 60004-1-Ig, Proteintech, Wuhan, China) or α-Tubulin (1:20,000, 66031-1-Ig, Proteintech) was used as the loading control.

### 4.14. Statistical Analysis

All experiments were performed with at least three independent biological replicates. Statistical analyses were performed using GraphPad Prism 8.0 (GraphPad Software, La Jolla, CA, USA). Data from three independent experiments are presented as mean ± SEM. Multiple comparisons were carried out using one-way analysis of variance (ANOVA). A *p* < 0.05 was considered statistically significant.

## 5. Conclusions

In conclusion, by integrating network pharmacology analysis with experimental validation, this study demonstrates that the anti-NSCLC effects of HP are primarily associated with the modulation of the cell cycle pathway. The key active component, Linarin, induces G0/G1 arrest, accompanied by the downregulation of hub targets such as Cyclin A2, which collectively contribute to the inhibition of proliferation, ultimately promoting tumor cellular senescence and apoptosis. Overall, our findings highlight the potential of HP and its active component Linarin as a feasible therapeutic strategy for NSCLC.

Nonetheless, this study has several limitations. The most significant limitation is the lack of in vivo validation, as our conclusions are currently based entirely on in silico and in vitro evidence. Future research must include animal studies, specifically by conducting in vivo studies using NSCLC xenograft mouse models to confirm the anti-tumor activity and safety profile of Linarin, coupled with pharmacokinetic analyses to understand its absorption, distribution, metabolism, and excretion properties in vivo. Furthermore, although molecular docking and Western blotting provided preliminary validation, the predictions from network pharmacology require further experimental confirmation. Future work should employ more direct methods, such as surface plasmon resonance (SPR) to quantitatively verify the binding affinity, and molecular dynamics simulations to evaluate the complex stability and interactions in a dynamic, solvated environment. Additionally, while this study primarily focused on Linarin, it may be interesting to determine whether other active components of HP also have anti-NSCLC effects, either individually or in synergy with Linarin. Finally, the specific mechanisms underlying the enhanced efficacy of Linarin in p53-deficient cells remain to be fully elucidated and represent an important direction for future research.

## Data Availability

The original contributions presented in this study are included in the article. Further inquiries can be directed to the corresponding authors.

## Abbreviations

The following abbreviations are used in this manuscript:

NSCLC	Non-small-cell lung cancer
SCLC	Small-cell lung cancer
LUAD	Lung adenocarcinoma
LUSC	Lung squamous cell carcinoma
TCM	Traditional Chinese medicine
HP	Herba Patriniae
CRC	Colorectal cancer
GEO	Gene Expression Omnibus
TCMSP	Traditional Chinese Medicine Systems Pharmacology Database
OB	Oral bioavailability
DL	Drug-likeness
DEGs	Differential expressed genes
PPI	Protein–protein interaction
GO	Gene Ontology
KEGG	Kyoto Encyclopedia of Genes and Genomes
BP	Biological processes
CC	Cellular components
MF	Molecular function
SA-β-gal	Senescence-associated β-galactosidase
SASP	senescence-associated secretory phenotype
NK cells	natural killer cells

## Appendix A

**Table A1:** Active components of HP.

Mol ID	Molecule Name	OB (%)	DL	CAS Number	Pubchem CID
MOL001676	Vilmorrianine C	33.96	0.22	73870-35-6	20055981
MOL001677	Asperglaucide	58.02	0.52	56121-42-7	10026486
MOL001678	Bolusanthol B	39.94	0.41	N/A	10594416
MOL001790	Linarin	39.84	0.71	480-36-4	5317025
MOL001689	Acacetin	34.97	0.24	480-44-4	5280442
MOL002322	Isovitexin	31.29	0.72	38953-85-4	162350
MOL001697	Sinoacutine	63.39	0.53	4090-18-0	821366
MOL000358	Beta-sitosterol	36.91	0.75	83-46-5	222284
MOL000359	Sitosterol	36.91	0.75	N/A	12303645
MOL000422	Kaempferol	41.88	0.24	520-18-3	5280863
MOL000449	Stigmasterol	43.83	0.76	83-48-7	5280794
MOL000006	Luteolin	36.16	0.25	491-70-3	5280445
MOL000098	Quercetin	46.43	0.28	117-39-5	5280343

**Table A2:** Hub genes of HP against NSCLC.

Hub Genes	Degree	Eigenvector	Betweenness	Closeness
CDK1	30	0.33105	100.2002	0.029294
CCNA2	30	0.334411	175.4002	0.029341
CCNB1	28	0.326259	164.9002	0.029329
CHEK1	12	0.122021	283.8788	0.029412
CDK4	12	0.098278	353.3	0.029459
CHEK2	8	0.058702	101.8667	0.029364
